# Deletion of p53 and Hyper-Activation of PIK3CA in Keratin-15^+^ Stem Cells Lead to the Development of Spontaneous Squamous Cell Carcinoma

**DOI:** 10.3390/ijms21186585

**Published:** 2020-09-09

**Authors:** Samantha M. Y. Chen, Li Bian, Andrew G. Nicklawsky, Alexandra L. Krinsky, Tonya Brunetti, Rachel A. Woolaver, Xiaoguang Wang, Zhangguo Chen, Christian D. Young, Dexiang Gao, Xiao-Jing Wang, Jing H. Wang

**Affiliations:** 1 Department of Immunology and Microbiology, University of Colorado Anschutz Medical Campus, School of Medicine, Aurora, CO 80045, USA; samantha.chen@cuanschutz.edu (S.M.Y.C.); alexandra.krinsky@cuanschutz.edu (A.L.K.); tonya.brunetti@cuanschutz.edu (T.B.); rachel.woolaver@cuanschutz.edu (R.A.W.); xiaoguang.wang@cuanschutz.edu (X.W.); zhangguo.chen@cuanschutz.edu (Z.C.); 2 Department of Pathology, University of Colorado Anschutz Medical Campus, School of Medicine, Aurora, CO 80045, USA; li.bian@cuanschutz.edu (L.B.); christian.young@cuanschutz.edu (C.D.Y.); 3 Department of Pediatrics, Department of Biostatistics and Informatics, Cancer Center Biostatistics Core, University of Colorado Anschutz Medical Campus, School of Medicine, Aurora, CO 80045, USA; andrew.nicklawsky@cuanschutz.edu (A.G.N.); dexiang.gao@cuanschutz.edu (D.G.); 4 Veterans Affairs Medical Center, VA Eastern Colorado Health Care System, Aurora, CO 80045, USA

**Keywords:** cancer immunotherapy, head and neck cancers, tumor microenvironment, tumor infiltrating lymphocytes

## Abstract

Squamous cell carcinoma (SCC) is the second commonest type of skin cancer, and SCCs make up about 90% of head and neck cancers (HNSCCs). HNSCCs harbor two frequent molecular alterations, namely, gain-of-function alterations of phosphatidylinositol-4,5-bisphosphate 3-kinase catalytic subunit alpha (*PIK3CA)* and loss-of-function mutations of tumor protein p53 (*TP53*). However, it remains poorly understood whether HNSCCs harboring different genetic alterations exhibit differential immune tumor microenvironments (TME). It also remains unknown whether *PIK3CA* hyperactivation and *TP53* deletion can lead to SCC development spontaneously. Here, we analyzed the Cancer Genome Atlas (TCGA) datasets of HNSCCs and found that patients with both *PIK3CA* and *TP53* alterations exhibited worse survival, significantly lower CD8 tumor infiltrating lymphocytes (TILs) and higher M0 macrophages than other controls. To better model human tumorigenesis, we deleted *TP53* and constitutively activated *PIK3CA* in mouse keratin-15-expressing stem cells, which leads to the spontaneous development of multilineage tumors including SCCs, termed Keratin-15-p53-PIK3CA (KPPA) tumors. KPPA tumors were heavily infiltrated with myeloid-derived suppressor cells (MDSCs), with a drastically increased ratio of polymorphonuclear-MDSC (PMN-MDSC) versus monocytic-MDSC (M-MDSC). CD8 TILs expressed more PD-1 and reduced their polyfunctionality. Overall, we established a genetic model to mimic human HNSCC pathogenesis, manifested with an immunosuppressive TME, which may help further elucidate immune evasion mechanisms and develop more effective immunotherapies for HNSCCs.

## 1. Introduction

Head and neck cancers (HNC) are a heterogeneous group of tumors arising from the mucosal surfaces of the upper aerodigestive tract [1]. Collectively, HNC is the sixth most prevalent cancer worldwide [1]. Some 90% of all HNCs are head and neck squamous cell carcinomas (HNSCCs) and HNSCCs are often associated with either carcinogens, such as alcohol and tobacco use, or oncogenic human papillomavirus (HPV) infection [2,3], thereby categorized as HPV(−) or HPV(+) HNSCCs. HNSCCs have been found to be diverse with a high rate of genetic heterogeneity, resulting in hyper-activation of oncogenes (e.g., *PIK3CA* and *HRAS*) and loss-of-function mutations in tumor suppressor genes (e.g., *TP53*, *CASP8*, and *NOTCH1*) [4,5]. Phosphoinositide 3-kinase (PI3K) is a frequently deregulated pathway in HNSCCs with a phosphatidylinositol-4,5-bisphosphate 3-kinase catalytic subunit alpha (*PIK3CA*) gene mutation rate of approximately 16% and gene amplification of more than 30% in tumors [6,7]. PI3Ks are activated by receptor tyrosine kinases (RTKs), such as epidermal growth factor receptor (EGFR), and consist of different classes of enzymes vital for differentiation, proliferation and cell survival [8]. Mammalian target of rapamycin (mTOR) complexes (mTORC1 and mTORC2) and protein kinase B (also known as AKT) are also involved in this pathway that can activate transcription and other signaling molecules of the PI3K pathway [9]. Monoclonal antibodies (mAbs) that inhibit EGFR have been used for both HPV(−) and HPV(+) subtypes of HNSCCs; however, they were found to have limited efficacy and elicited resistance [10].

Another highly mutated gene in HNSSCs is the tumor protein p53 (*TP53*) gene, with over 80% of HPV(−) HNSCCs harboring loss-of-function mutations in *TP53*; however, *TP53* mutations occur much less frequently in HPV(+) HNSCCs (~3%) [4]. *TP53* is a tumor-suppressor gene encoding a transcription factor that maintains DNA repair, cell cycle, senescence and apoptosis [11]. These attributes make p53 an important cell sensor for oncogene activation and DNA damage. It has been found that the degradation of p53 is associated with HPV E6 oncoproteins [3]. Although there have been several therapies that target p53 in hopes to restore p53 function, they have yet to be proven effective in clinical trials [12]. By and large, *TP53* mutations are associated with poor HNSCC prognosis and overall survival with increased rate of recurrence and resistance to therapies. It remains poorly understood whether HNSCCs harboring different genetic alterations exhibit differential immune tumor microenvironment (TME). For instance, it is unknown whether HNSCCs with the double mutations in *TP53* and *PIK3CA* have a more immunosuppressive TME.

Prior studies have generated murine models that mimicked the alterations of *PIK3CA* or *p53* in HNSCCs. Transgenic mice that overexpressed wild-type PIK3CA in head and neck epithelium were generated; however, PIK3CA overexpression alone was not sufficient to initiate HNSCC formation [6]. Nevertheless, these PIK3CA Tg mice were much more susceptible to 4-nitroquinoline 1 oxide (4NQO)-induced HNSCC carcinogenesis [6]. Conditional deletion of p53 in mouse epithelial cells with K14.CrePR1 led to SCC development in about half of mice after 20 months [13]. To establish a mouse model that more closely resembles the genetic alterations in HNSCCs and allows us to better investigate immune evasion mechanisms of HNSCCs, we generated a novel genetic model by deleting *p53* and constitutively activating *PIK3CA* in mouse keratin 15-expressing (K15^+^) stem cells, which leads to the development of multilineage tumors including SCCs, termed Keratin-15-p53-PIK3CA (KPPA) tumors. We found that the TME of KPPA tumors appeared to be highly immunosuppressive. We suggest that the KPPA tumor model may help further elucidate immune evasion mechanisms in HNSCCs and develop more effective HNSCC immunotherapies.

## 2. Results

### 2.1. HNSCC Patients with Double Genetic Alterations in PIK3CA and TP53 Exhibited Worse Prognosis and More Immunosuppressive TME

**Figure 1:**
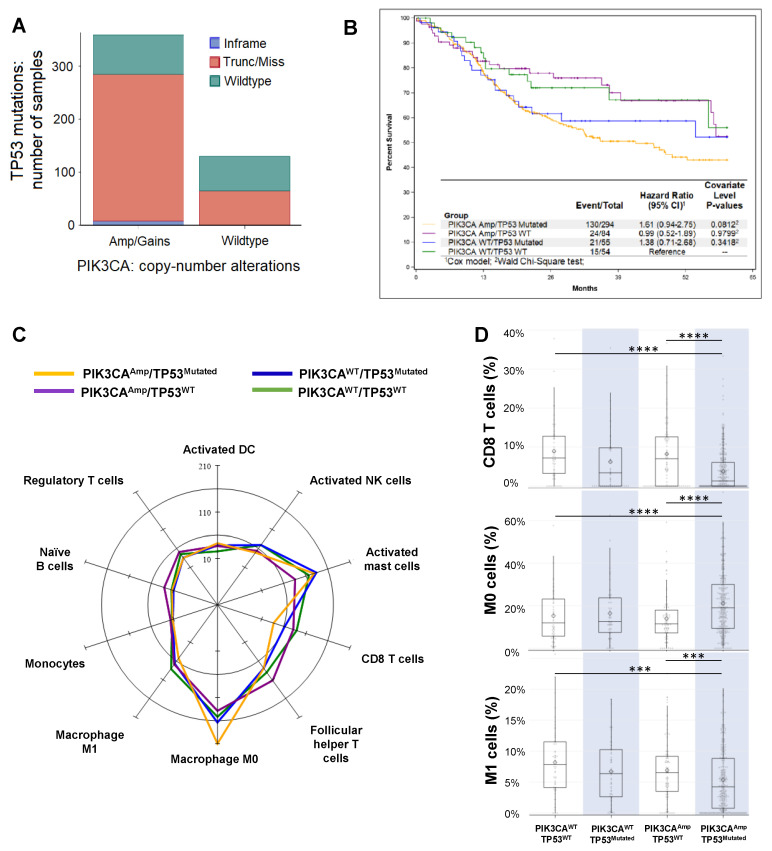
Analysis of The Cancer Genome Atlas (TCGA) datasets of Head and Neck Squamous cell carcinoma (HNSCC) patients. (**A**) Association between PIK3CA gene alterations and TP53 gene mutation in HNSCC patients (TCGA, PanCancer Atlas, *n* = 489 samples). The Cancer Genome Atlas (TCGA) RNA-seq data and clinical data of HNSCC cohorts were obtained from the cBioPortal (https://cbioportal.org) (see details in Method). Patients with PIK3CA^Amp/gain^ (*n* = 359) had a higher chance to harbor TP53 mutations than patients with PIK3CA^WT^ (*n* = 130). (**B**) Kaplan-Meier overall 5-year survival curves of HNSCC patients in 4 groups (PIK3CA^Amp^/TP53^Mutated^, PIK3CA^Amp^/TP53^WT^, PIK3CA^WT^/TP53^Mutated^, and PIK3CA^WT^/TP53^WT^) as described in Methods. Only patients with available survival data were included for this analysis (*n* = 487). (**C**) A radar plot of the cell types that reached significance in the omnibus Kruskal-Wallis test when comparing among the 4 groups. The scale is per 1000 cells. (**D**) Box and whisker plots of CD8 T cells, resting macrophages (M0), and M1 macrophages. The expression of CD8 T cell signature genes: PIK3CA^Amp^/TP53^Mutated^ group (3.77 ± 5.32) was significantly lower (*p* < 0.0001) than groups of PIK3CA^WT^/TP53^WT^ (8.97 ± 7.86) and PIK3CA^Amp^/TP53^WT^ (8.22 ± 8.12). The expression of M0 signature genes: PIK3CA^Amp^/TP53^Mutated^ group (21.07 ± 14.08) was significantly higher (*p* < 0.0001) than groups of PIK3CA^WT^/TP53^WT^ (15.25 ± 12.32) and PIK3CA^Amp^/TP53^WT^ (13.93 ± 11.23). The expression of M1 signature genes: PIK3CA^Amp^/TP53^Mutated^ group (5.35 ± 5.05) was significantly lower (*p* < 0.0002) than groups of PIK3CA^WT^/TP53^WT^ (8.17 ± 5.62) and PIK3CA^Amp^/TP53^WT^ (6.92 ± 5.17).

The Cancer Genome Atlas (TCGA) RNA-seq data and clinical data of HNSCC cohorts were obtained from the cBioPortal (https://cbioportal.org) (see details in Methods). In analyzing the dataset of HNSCC patients (TCGA, PanCancer Atlas, *n* = 489 samples), we found that the patients with *PIK3CA* alterations, including amplification and gain, also have a higher chance of harboring *TP53* mutations (Figure 1A). After merging two datasets (see details in Methods), we divided the patients into four different groups: PIK3CA^Amp^/TP53^Mutated^ (*n* = 294), PIK3CA^Amp^/TP53^WT^ (*n* = 85), PIK3CA^WT^/TP53^Mutated^ (*n* = 56), and PIK3CA^WT^/TP53^WT^ (*n* = 54). Kaplan Meier curves of five-year survival were shown for four different groups, and PIK3CA^Amp^/TP53^Mutated^ group exhibited a hazard ratio of 1.61 (95% CI, 0.94–2.75) compared to PIK3CA^WT^/TP53^WT^ group (Figure 1B). PIK3CA^Amp^/TP53^Mutated^ group’s 10-year survival hazard ratio was 1.8 (95% CI, 1.06–3.07), and showed a significantly worse prognosis than PIK3CA^WT^/TP53^WT^ group (Supplemental Figure S1).

We uploaded RNA-seq data of HNSCC patients onto CIBERSORT (see details in Methods), which estimated the relative proportions of 22 immune cell types (Supplemental Figure S2), with a more in-depth dissection shown in Supplemental Table S1. Both innate and adaptive immune cells varied in their expression levels depending on the genetic alterations in 4 groups (Figure 1C). In particular, we found that the expression of CD8 T cell signature genes was significantly lower in PIK3CA^Amp^/TP53^Mutated^ group, compared with PIK3CA^WT^/TP53^WT^ and PIK3CA^Amp^/TP53^WT^ groups (Figure 1D). In addition, PIK3CA^Amp^/TP53^Mutated^ group had significantly lower expression of activated NK cell-associated genes compared with PIK3CA^WT^/TP53^WT^ and PIK3CA^WT^/TP53^Mutated^ groups (Supplemental Table S1). PIK3CA^Amp^/TP53^Mutated^ group expressed significantly higher level of resting macrophage (M0) signature genes but lower level of activated macrophage (M1) genes than PIK3CA^WT/^TP53^WT^ and PIK3CA^Amp^/TP53^WT^ groups (Figure 1D). We conclude that HNSCCs with the genotype of PIK3CA^Amp^/TP53^Mutated^ appear to have a highly immunosuppressive TME.

### 2.2. A Genetic Mouse Model of PIK3CA Hyperactivation and p53 Deletion in K15^+^ Cells

**Figure 2:**
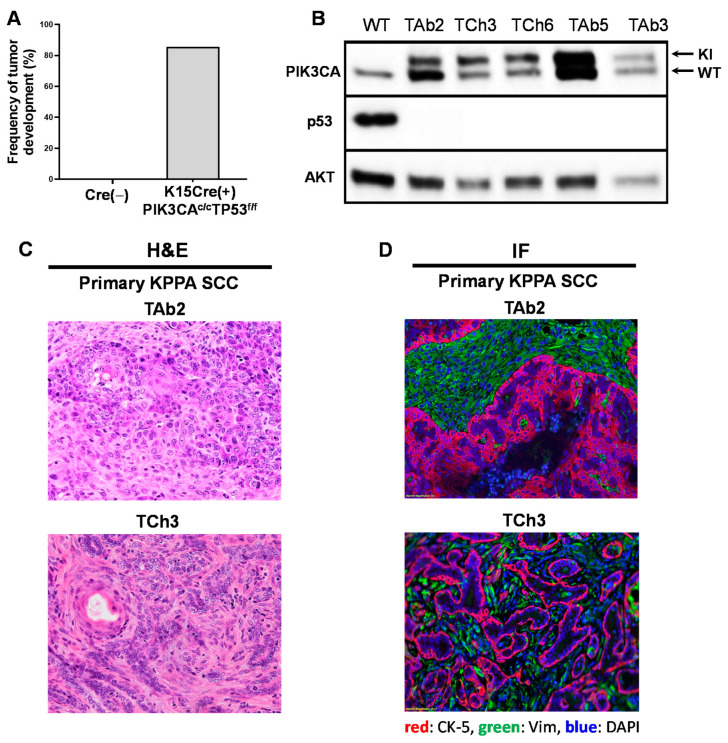
Establishment of Keratin-15-p53-PIK3CA (KPPA) tumor models by deleting tumor protein p53 (*TP53*) and constitutively activating phosphatidylinositol-4,5-bisphosphate 3-kinase catalytic subunit alpha (*PIK3CA*). (**A**) Bar graph showing the frequency of tumor development. None of the littermate control mice (K15.CrePR1(−)*PIK3CA^C/C^/TP53^f/f^*) developed tumors (*n* = 10). Of the K15.CrePR1(+)*PIK3CA^C/C^/TP53^f/f^* mice, 85.7% developed tumors (*n* = 14); (**B**) Western blot analysis showing the deletion of p53 and expression of constitutively active PIK3CA protein in KPPA tumors. Five independent KPPA tumors were analyzed by Western blot with wild-type (WT) B6 spleen as control. KPPA tumors expressed both endogenous (lower band) and knock-in (KI) (higher band) PIK3CA protein, and deleted p53 protein. AKT protein is the loading control; (**C**) H&E analysis of KPPA tumor morphology. Representative images of H&E staining for primary KPPA squamous cell carcinomas (SCCs) and primary KPPA pleomorphic carcinoma; (**D**) Immunofluorescence (IF) staining of KPPA tumors. Representative images of IF staining for primary KPPA SCCs and primary KPPA pleomorphic carcinoma. Cytokeratin 5 (CK-5^+^) (red) tumor cells were separated from vimentin (Vim^+^) (green) stroma cells. DAPI (blue) staining indicated nuclei. Magnification: 20×.

To establish a mouse model that can mimic the two most frequently mutated genes in human HNSCCs, we crossed a K15.CrePR1 transgenic mouse model that expressed a RU486-inducible Cre recombinase in K15^+^ bulge epithelial stem cells with a floxed *trp53* allele (p53^f/f^) and a knock-in allele of the constitutively active *pik3ca* gene in the ROSA26 locus (PIK3CA^c/c^) to generate K15.CrePR1(+)p53^f/f^PIK3CA^c/c^ mice. RU486 application causes the activation of homozygous *PIK3CA^c/c^* knock-in allele and conditional deletion of *trp53* in K15^+^ epithelial stem cells. Upon RU486 application, we observed that none of the mice in the Cre^−^ cohort developed tumors, whereas, a majority of K15.CrePR1(+)p53^f/f^PIK3CA^c/c^ mice developed cutaneous tumors 2−3 months after induction of Cre-mediated recombination, termed KPPA tumors (Figure 2A, Supplemental Table S2). Individual mice developed a varying number of tumors (ranging from one to multiple tumors per mouse), which is likely due to the efficiency of Cre-mediated recombination. 

We analyzed the primary KPPA tumors by Western blotting to examine the expression of p53 and PIK3CA (a.k.a. p110α) proteins. Our data demonstrated that the primary KPPA tumors did not express p53 (Figure 2B), and harbored the constitutively active *PIK3CA* allele that encoded a protein with a slightly higher molecular weight than the WT PIK3CA protein (Figure 2B). Of note, we still detected the WT PIK3CA protein in all of the KPPA tumors (Figure 2B), because the WT endogenous *PIK3CA* locus remained intact. Based on H&E histological assessment, KPPA tumors were characterized as either well-to-moderately differentiated SCC or pleomorphic carcinoma (Figure 2C). Immunofluorescent staining of cytokeratin 5 (CK-5) and vimentin (Vim) confirmed these results (Figure 2D). Overall, we conclude that deleting p53 and constitutively activating PIK3CA in mouse K15-expressing stem cells leads to the development of multilineage tumors including SCCs.

### 2.3. Characterization of the Immune TME in KPPA Tumors

To better understand how KPPA tumors evaded the host’s immunity, we performed flow cytometry analysis to characterize different subsets of immune cells in KPPA TME. As controls, we analyzed the splenocytes collected from either wildtype (WT) B6 mice or mice harboring KPPA tumors that spontaneously arose upon RU486 induction. To test whether tumors harboring different oncogenic drivers exhibit differential immune profiles in the TME, we also transplanted a SCC line (A223) [14] derived from primary K15.Kras^G12D^.Smad4^−/−^ SCCs [15] into WT B6 recipient mice and analyzed the immune cells in these A223 tumors.

**Figure 3:**
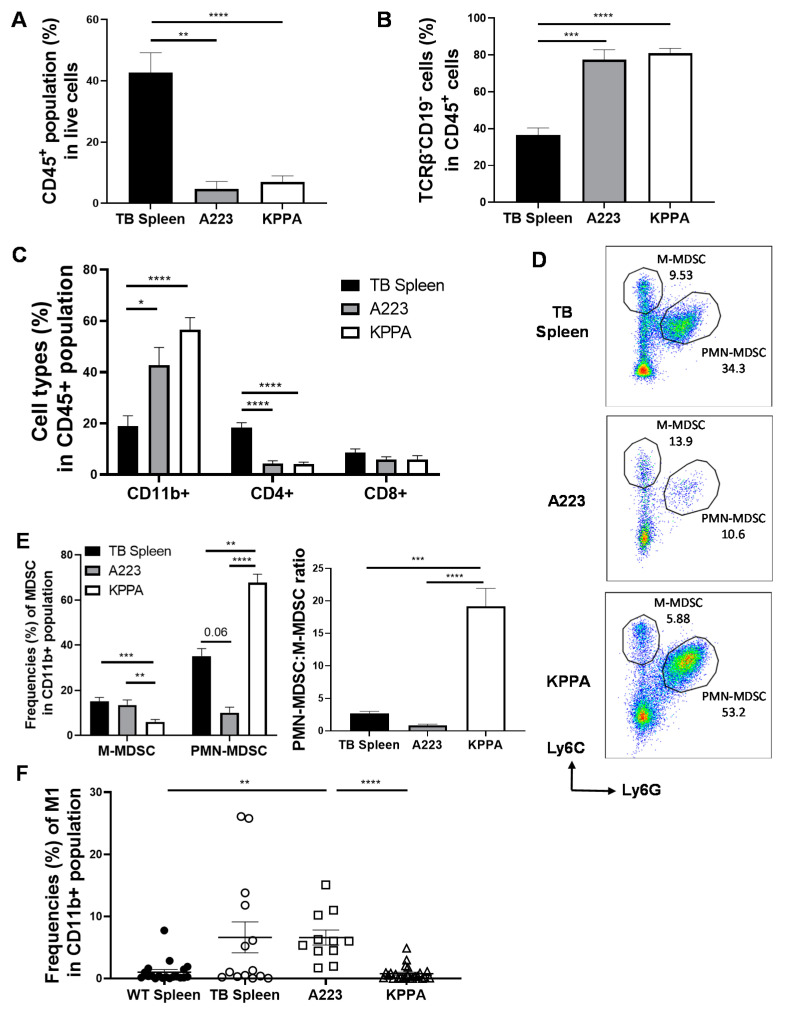
Characterization of the immune tumor microenvironment (TME) of KPPA tumors. Flow cytometry were performed for tumor-bearing (TB) splenic control (*n* = 14), or the TILs from A223 (*n* = 11) and KPPA (*n* = 25) tumors for all panels shown below. (**A**) Quantification of the percentage of total CD45^+^ hematopoietic population. The percentage of CD45^+^ population was significantly greater in TB splenic control (42.62 ± 6.50) than A223 (*p* = 0.0014) or KPPA (*p* ≤ 0.0001) tumors; (**B**) Quantification of the percentage of CD45^+^TCRβ^−^CD19^−^ population (non-T and non-B cells). The percentage of CD45^+^TCRβ^−^CD19^−^ population was significantly lower in TB splenic control (36.47 ± 3.86) than A223 (77.44 ± 5.43, *p* = 0.0004) or KPPA (80.94 ± 2.69, *p* ≤ 0.0001) tumors; (**C**) Quantification of the percentage of CD11b^+^, CD4^+^, or CD8^+^ cells in CD45^+^ population of TB spleen, A223 or KPPA tumors. For CD11b^+^ cells: TB splenic control (18.94 ± 3.99) was significantly lower than A223 (42.11 ± 6.84, *p* = 0.05) and KPPA (56.57 ± 4.70, *p* ≤ 0.0001) tumors. For CD4^+^ cells: A223 (4.26 ± 1.13, *p* ≤ 0.0001) and KPPA (4.20 ± 0.61, *p* ≤ 0.0001) tumors were significantly lower than TB splenic control (17.43 ± 2.00). For CD8^+^ cells: no significant differences between KPPA tumors (5.92 ± 1.44), A223 tumors (5.87 ± 1.05) and TB splenic control (7.92 ± 1.49); (**D**) Representative flow plots for different MDSC populations. M-MDSC (CD11b^+^Ly6G^−^Ly6C^high^) vs. PMN-MDSC (CD11b^+^Ly6G^+^Ly6C^low^); (**E**) **Left panel:** Quantification of the percentage of M-MDSC vs. PMN-MDSC in indicated groups. There was no difference in M-MDSC between TB spleen (15.12 ± 1.75) and A223 tumors (13.49 ± 2.22). The percentage of M-MDSC was significantly lower in KPPA tumors (6.07 ± 1.02) than TB spleen (*p* ≤ 0.0001) or A223 tumors (*p* = 0.018). The percentage of PMN-MDSC was significantly higher in KPPA tumors (67.74 ± 3.72) than TB spleen (35.14 ± 3.32, *p* = 0.002) or A223 tumors (9.78 ± 2.53, *p* ≤ 0.0001). The difference of PMN-MDSC population between TB spleen and A223 tumors is not statistically significant (*p* = 0.06). **Right panel:** The ratio of PMN-MDSCs vs. M-MDSCs in indicated groups. PMN-MDSC vs. M-MDSC ratio in KPPA tumors (19.42 ± 1.64) was significantly higher than TB splenic control (2.68 ± 0.33, *p* = 0.0005) and A223 tumors (0.81 ± 0.18, *p* ≤ 0.0001); (**F**) Analysis of classically activated macrophage (M1) population. The percentage of M1 in CD11b^+^ population was significantly higher in A223 tumors (6.59 ± 1.21) than WT splenic control (1.02 ± 0.41, *p* = 0.0013) or KPPA tumors (1.88 ± 1.13, *p* = 0.0003). Data were analyzed using Kruskal-Wallis test * *p* < 0.05, ** *p* < 0.01, *** *p* < 0.001, **** *p* < 0.0001, with Dunn’s multiple-comparison test correction.

To differentiate hematopoietic cells from other cell lineages, we performed flow analysis on the single-cell suspension of the WT spleen control, KPPA tumor-bearing (TB) spleen control, A223 and KPPA tumors, and gated on the CD45^+^ population (a marker for hematopoietic cells). We found that the percentage of CD45^+^ cells was significantly less in both A223 and KPPA tumors compared to both splenocyte controls, while the percentage of CD45^+^ cells did not differ in A223 and KPPA tumors (Figure 3A, Supplemental Figure S3A). Within the CD45^+^ population, we also identified the non-B cell/non-T cell (TCRβ^−^CD19^−^) population in these samples and found that both A223 and KPPA tumors harbored a significantly higher percentage of TCRβ^−^CD19^−^ population than the splenic controls (Figure 3B, Supplemental Figure S3B). Further classification of the tumor-infiltrating immune cells showed that both SCC tumor models exhibited a much higher percentage of myeloid population (TCRβ^−^CD19^−^CD11b^+^) compared to splenic controls (Figure 3C, Supplemental Figure S3C). We also examined the tumor infiltrating lymphocytes (TILs) including CD4 and CD8 T cells and found that both A223 and KPPA tumors contained a much lower percentage of CD4 T cell than splenic controls (Figure 3C, Supplemental Figure S3C). While the percentage of CD8 T cells did not differ between KPPA tumors and the TB splenic control (Figure 3C), it was significantly lower in KPPA tumors than in the WT splenic control (Supplemental Figure S3C). The difference in the percentage of cell type between WT and TB spleens may be due to metastases in the TB spleens, as indicated by the higher percentage of non-CD45 population (Supplemental Figure S4, TB-Spleen 54.8% vs. WT-spleen 2.08%). Taken together, we concluded that both SCC tumors (KPPA and A223) were heavily infiltrated by myeloid cell populations (CD11b^+^) but not by CD4 or CD8 TILs.

It has been established that the two subsets of myeloid-derived suppressor cells (MDSCs), namely, M-MDSC (monocytic-MDSC) defined as CD11b^+^Ly6G^−^Ly6C^high^ and PMN-MDSC (polymorphonuclear-MDSC) defined as CD11b^+^Ly6G^+^Ly6C^low^, play a pivotal role in immune suppression during tumorigenesis [16]. By gating on the CD11b^+^ population with gating strategies established previously (Supplemental Figure S5) [16,17], we examined the percentage of M-MDSC vs. PMN-MDSC in both SCC tumors and splenic controls (Figure 3D,E, Supplemental Figure S3D). We found that the percentage of M-MDSC did not differ between the TB spleen and A223 tumors (Figure 3E). In contrast, the percentage of M-MDSC was significantly reduced in KPPA tumors compared to A223 tumors or both splenic controls (Figure 3E, Supplemental Figure S3D). Similarly, there were no statistically significant differences between the percentage of PMN-MDSC in splenic controls and A223 tumors (Figure 3E, Supplemental Figure S3D). Remarkably, KPPA tumors had a significantly higher percentage of PMN-MDSC than both the TB spleen and A223 tumors (Figure 3E). The ratio of PMN-MDSC vs. M-MDSC was drastically increased in KPPA tumors compared with other controls (Figure 3E, Supplemental Figure S3E). Thus, KPPA tumors exhibit a preferential increase of PMN-MDSC phenotype. Our analysis of TCGA human patient cohorts showed that PIK3CA^Amp^/TP53^Mutated^ group expressed significantly lower levels of signature genes for M1 population than PIK3CA^WT/^TP53^WT^ and PIK3CA^Amp^/TP53^WT^ groups (Figure 1D). Since tumor-associated macrophages may be an important aspect to the TME, we examined the classically activated macrophage (M1) population using the gating strategy published by previous studies [18,19,20,21] (Supplemental Figure S5). Our data showed that KPPA tumors exhibited a significantly lower frequency of M1 population than A223 tumors (Figure 3F). Taken together, these results are consistent with the notion that KPPA tumors present an immunosuppressive TME.

### 2.4. Dysfunctional TILs in KPPA Tumors Suggest an Immunosuppressive TME

**Figure 4:**
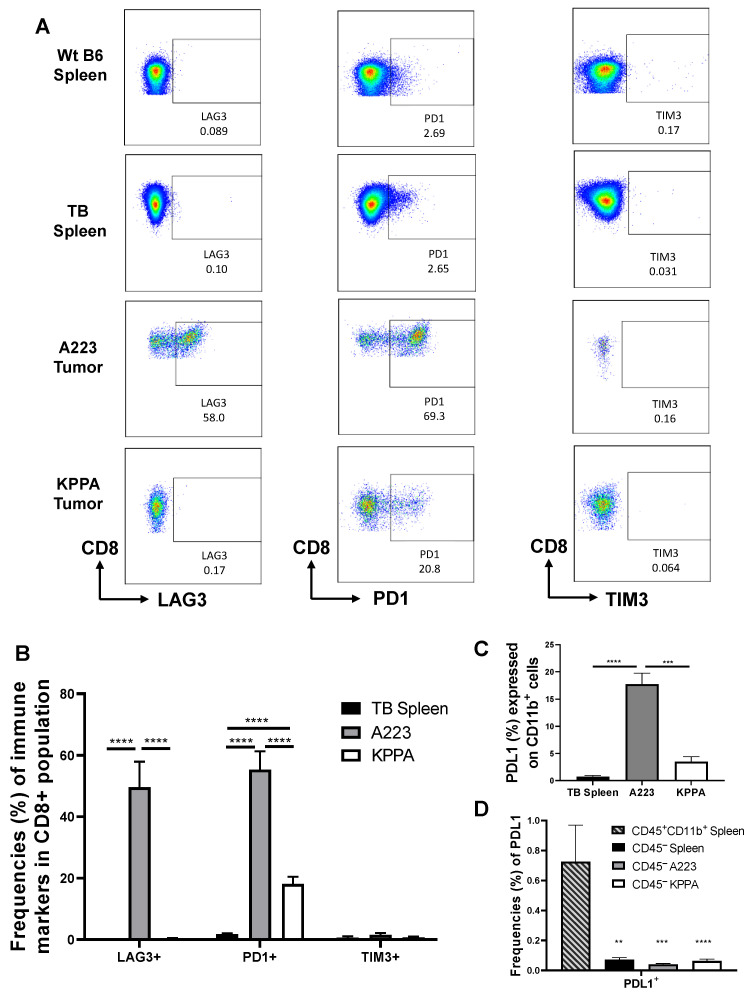
Expression of checkpoint molecules in CD8 T cells and programmed cell death 1-ligand 1 (PD-L1) expression in different types of cells. (**A**) Representative flow plots for lymphocyte-activation gene 3 (LAG-3), T cell immunoglobulin and mucin domain 3 (TIM-3) and PD-1 expression on CD8 T cells in WT splenic control, TB splenic control, A223 and KPPA tumors; (**B**) Quantification of the percentage of CD8 T cells expressing different markers in TB splenic control (*n* = 14), A223 (*n* = 11) and KPPA tumors (*n* = 24); The percentage of LAG-3^+^CD8^+^ population was significantly higher in A223 tumors (49.61 ± 8.3) than KPPA tumors (0.29 ± 0.18, *p* ≤ 0.0001) and TB splenic control (0.10 ± 0.04, *p* ≤ 0.0001); The percentage of PD1^+^CD8^+^ population was significantly increased in KPPA tumors (18.09 ± 2.34, *p* = 0.0016) and A223 tumors (55.32 ± 5.98, *p* ≤ 0.0001) compared with the TB splenic control (2.39 ± 0.70), and was significantly higher in A223 tumors than KPPA tumors (*p* ≤ 0.0001). No difference was observed for TIM-3^+^CD8^+^ population in all groups; (**C**) Quantification of the percentage of PD-L1^+^CD11b^+^ population in indicated groups. Statistically significant differences were observed between A223 tumors (17.70 ± 2.03) and TB splenic control (0.72 ± 0.24, *p* ≤ 0.0001) or between A223 and KPPA tumors (3.49 ± 0.94, *p* = 0.0001); (**D**) Quantification of the percentage of PD-L1^+^ in indicated groups. Statistically significant differences were observed between CD45^+^CD11b^+^ spleen and all other CD45^−^ groups. No statistical differences observed between all CD45^−^ groups including TB spleen (0.072 ± 0.013), A223 tumors (0.04 ± 0.006) and KPPA tumors (0.063 ± 0.012). Data were analyzed using Kruskal-Wallis test ** *p* < 0.01, *** *p* < 0.001, **** *p* < 0.0001, with Dunn’s multiple-comparison test correction.

To assess the expression level of immune checkpoint molecules, we performed flow cytometry analysis by comparing WT or TB splenic control, and CD8 TILs from A223 or KPPA tumors. We found that both WT and TB splenic CD8 T cells expressed a similar level of checkpoint molecules, lymphocyte-activation gene 3 (LAG-3), programmed cell death 1 (PD-1), and T cell immunoglobulin and mucin domain 3 (TIM-3) (Figure 4A), thus, we compared the CD8 TILs to TB splenic control (Figure 4B). CD8 T cells in all groups expressed a negligible level of TIM-3 (Figure 4A,B). Only CD8 TILs in A223 tumors expressed a high level of LAG-3 [14], while CD8 T cells in other groups did not (Figure 4A,B). Of note, we observed that CD8 TILs in KPPA tumors expressed a much higher level of PD-1 compared with the splenic CD8 T cells, while CD8 TILs in A223 tumors expressed the highest level of PD-1 (Figure 4A,B).

Next, we measured the expression of PD-L1 in different types of cells (Figure 4C,D). The percentage of CD11b^+^PD-L1^+^ population was significantly higher in A223 tumors than the TB spleens or KPPA tumors, while there was no statistically significant difference between the TB spleens and KPPA tumors (Figure 4C). With further examination of the PD-L1^+^ population in CD45^−^ cells, our data showed that all of the CD45^−^ populations expressed a minimal level of PD-L1 (<0.1%), including CD45^−^ cells from the TB spleens, A223 tumors and KPPA tumors, which was significantly lower than that in CD45^+^CD11b^+^ population in the TB spleens (Figure 4D).

**Figure 5:**
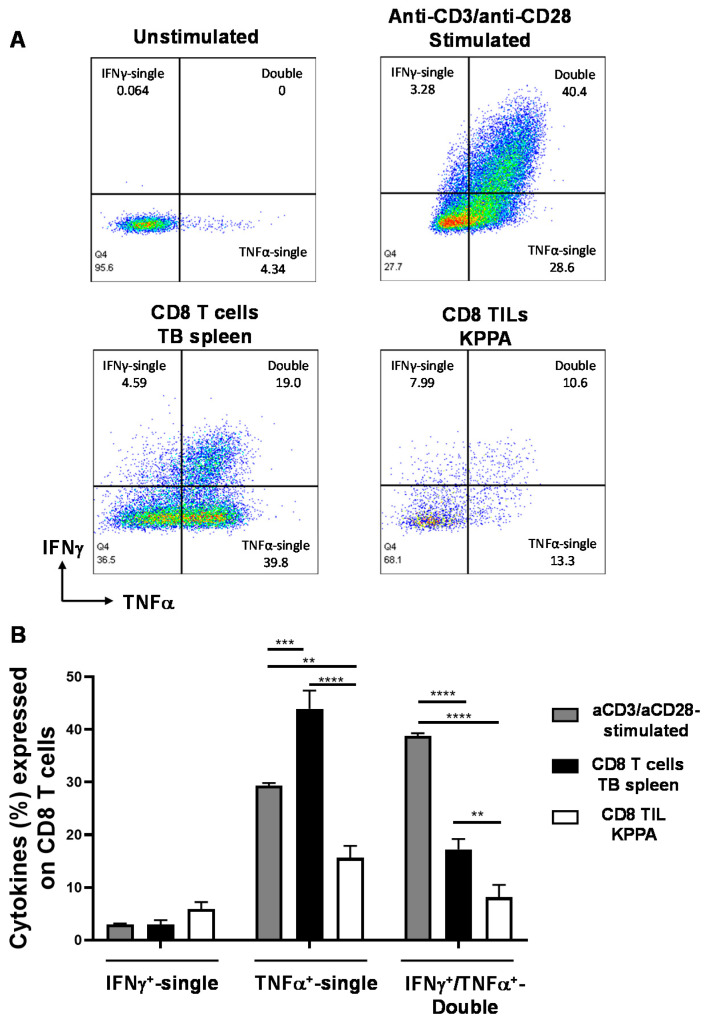
Reduced polyfunctionality of CD8 tumor-infiltrating lymphocytes (TILs) in KPPA tumors. (**A**) Representative flow plots of the CD8 T cells producing either single or double cytokines (IFNγ^+^-single, TNFα^+^-single, and IFNγ^+^TNFα^+^-double); (**B**) Quantification of the percentage of IFNγ^+^-single, TNFα^+^-single, and IFNγ^+^TNFα^+^-double in CD8 T cells of indicated groups. Intracellular cytokine staining was performed for the following groups: anti-CD3/anti-CD28 stimulated T cells (*n* = 6), CD8 T cells from TB spleen (*n* = 14) or CD8 TILs from KPPA tumors (*n* = 18). No statistical difference was observed when comparing IFNγ^+^-single production in CD8 T cells between anti-CD3/anti-CD28 stimulated (3.02 ± 0.14), the TB spleen (2.97 ± 0.84) and KPPA tumors (5.91 ± 1.32). Both groups of KPPA tumors (15.63 ± 2.26, *p* < 0.0001) and anti-CD3/anti-CD28 stimulated (29.33 ± 0.51, *p* = 0.0008) produced significantly lower levels of TNFα^+^-single than TB spleen group (43.92 ± 3.45). CD8 TILs from KPPA tumors (8.12 ± 2.36) produced significantly lower levels of IFNγ^+^TNFα^+^-double than TB spleen (17.22 ± 1.98, *p* = 0.0086) or anti-CD3/anti-CD28 stimulated (38.75±0.55, *p* < 0.0001). Percentages of cells were compared by two-way ANOVA with Tukey’s multiple-comparison test correction. ** *p* < 0.01, *** *p* < 0.001, **** *p* < 0.0001.

IFN-γ and TNF-α are commonly examined cytokines for evaluating T cell effector functions, especially for the polyfunctionality of T cells, which means T cells can produce not only one cytokine but also additional different cytokines. Polyfunctional T cells are effector T cells that retain cytotoxic potential and may be more effective in tumor suppression [22,23]. In addition, the loss of double producers (IFNγ^+^TNFα^+^) is often considered as a sign of CD8 T cell dysfunction [24]. To examine the functional changes in CD8 TILs of KPPA tumors, we performed intracellular cytokine staining by flow cytometry to detect the intracellular level of single IFN-γ^+^, single TNFα^+^ or double IFN-γ^+^TNFα^+^ production in CD8 T cells from different groups. As a negative control, unstimulated naïve CD8 T cells did not produce much cytokine (unstimulated) (Figure 5A). As a positive control, we stimulated CD8 T cells from WT B6 mice with anti-CD3/anti-CD28 beads for 3 days, then cultured these cells in the presence of PMA/ionomycin/BFA for 4-6 h, and examined the IFN-γ and TNF-α level. As shown in Figure 5A,B, the anti-CD3/anti-CD28 stimulated CD8 T cells contained the highest level of double producers (IFNγ^+^TNFα^+^), indicating a robust polyfunctionality and strong effector functions. Then, we compared the splenic CD8 T cells from tumor-bearing mice (CD8 T cells-TB spleen) and the CD8 TILs from KPPA tumors (CD8 TILs-KPPA). A vast majority of splenic CD8 T cells are naïve CD8 T cells and it has been shown that naïve CD8 T cells tended to produce a high level of TNF-α upon stimulation [25]. Consistently, we found that the percentage of single TNF-α^+^ CD8 T cells was the highest in the splenic CD8 T cells from tumor-bearing mice (CD8 T cells-TB Spleen) (Figure 5A,B). In contrast, we found that CD8 TILs from KPPA tumors exhibited the lowest level of effector functions, evidenced by a lower percentage of double producer (IFNγ^+^TNFα^+^) in CD8 TILs than not only anti-CD3/anti-CD28-stimulated but also splenic naïve CD8 T cells (Figure 5A,B). We found that there was no statistically significant difference in the percentage of single IFN-γ^+^ CD8 T cells in different groups (Figure 5B). Taken together, these results are consistent with the notion that CD8 TILs in KPPA tumors were exhausted with impaired effector functions.

## 3. Discussion

Comprehensive genomic and epigenetic analyses and flow cytometry-based assay of HNSCC samples demonstrate the heterogeneity in HNSCC molecular signature and immune landscape [4]. Our study further examined this premise and grouped the HNSCC patient cohort based on their genetic alterations of *TP53* and *PIK3CA*. We found that the patients with both *TP53* and *PIK3CA* gene alterations have a significantly greater hazard ratio and worse OS in 5 years. Consistent with previous studies that used CD8 T cell as a prognostic biomarker [26], we found that PIK3CA^Amp^/TP53^Mutated^ HNSCC patients expressed significantly lower levels of CD8 T cell gene signature in their tumor biopsy than PIK3CA^WT^/TP53^WT^ and PIK3CA^Amp^/TP53^WT^ patients (Figure 1D). Of note, we also found significantly higher expression levels of M0 subset and lower levels of M1 subset in PIK3CA^Amp^/TP53^Mutated^ group than PIK3CA^WT^/TP53^WT^ and PIK3CA^Amp^/TP53^WT^ (Figure 1D). Overall, we suggest that identifying a correlation between specific genetic and molecular signatures and immune TME may help to predict the clinical outcomes and provide potential therapeutic targets.

Our current study addresses a previously recognized limitation in the field, which is the inadequate preclinical models mimicking human HNSCCs characterized with specific genetic alterations, thereby hindering studies to further elucidate the mechanistic link between the immune TME and oncogenic drivers in SCCs and to develop new immunotherapies. In this regard, a recent study reported the establishment of a syngeneic mouse HNSCC model induced by 4-NQO that resembles the human tobacco-related HNSCC mutanome, including mutations in *TP53* and *FAT3* but not in *PIK3CA* [27]. To our knowledge, we first showed that genetic mutations of both *PIK3CA* hyper-activation and *TP53* deletion in K15^+^ stem cells resulted in spontaneous development of KPPA tumors including SCCs. Consistent with findings in TCGA HNSCC patients of PIK3CA^Amp^/TP53^Mutated^ cohort, we found KPPA tumors harbored a low level of CD8 TILs that expressed a higher level of PD-1 and exhibited reduced polyfunctionality, suggesting that these CD8 TILs were chronically activated and experienced exhaustion. However, the CD8 TILs in KPPA tumors did not express TIM-3 or LAG-3, an observation different from our previous studies of a different SCC model in which the CD8 TILs coexpressed a high level of PD-1 and LAG-3 [14]. These data suggest that the immune phenotypes of CD8 TILs vary and may be influenced by tumor cells with different genetic alterations or differentiation status. Although anti-PD-1/PD-L1 have been approved by FDA for treating HNSCCs, the overall response rate is still below 20% [28]. Various clinical studies of treating HNSCC patients with anti-PD-1/PD-L1 in combination with targeted therapy, radiation and chemotherapy are ongoing [28]. By further developing syngeneic transplanted mouse models of KPPA tumors, we may be able to provide a platform for better understanding the immune TME and developing combinatorial treatment and predictive markers for clinical outcomes of immunotherapies.

Notably, KPPA tumors were heavily infiltrated with MDSCs that exhibited a drastic increase in the ratio of PMN-MDSC versus M-MDSC. VEGF and TGF-β have been reported to recruit MDSCs into the TME and affect their differentiation [29]; however, it remains to be addressed how PMN-MDSCs were preferentially increased in KPPA tumors. Treating SCC with anti-CD11b monoclonal antibodies has been shown to prevent the recruitment of myeloid cells into tumors, which attenuates tumor growth, and enhances antitumor response to radiation [30]. PD-L1 expression on myeloid cells can induce T cell exhaustion and reduce the efficacy of T cell-associated immunotherapy in solid tumor [28]. Thus, anti-PD-L1 in combination with myeloid-targeted therapy may likely lead to better responses to T cell-mediated immunotherapy. In this regard, by generating new KPPA SCC lines, it will allow us to better investigate the mechanisms and responses to combinational targeted therapy for HNSCCs and beyond.

## 4. Materials and Methods

### 4.1. Analysis of Patient Samples Obtained by TCGA

The Cancer Genome Atlas (TCGA) RNA-seq data and clinical data of HNSCC cohorts were obtained from the cBioPortal (https://cbioportal.org). The TCGA datasets provided comprehensive genomic sequencing and signatures which allowed the identification of patients with *TP53* mutations and *PIK3CA* copy number changes. Within cBioPortal and under the category of head and neck cancers, we downloaded data from two cohorts of HNSCCs (TCGA, Firehose Legacy, *n* = 528 samples; and TCGA, PanCancer Atlas, *n* = 489 samples). We used the second HNSCC dataset (TCGA, PanCancer Atlas, *n* = 489 samples) for analysis in Figure 1A. These two data sets were merged utilizing patient IDs (*n* = 528) and used for analysis in Figure 1B–D; however, we only had 489 analyzable records due to 39 having missing group information (*n* = 489). Patients were divided into four different groups: (1) amplification and gain of *PIK3CA* copy number (PIK3CA^Amp^) and truncation and missense of *TP53* gene (TP53^Mutated^) (*n* = 294); (2) PIK3CA^Amp^ and wildtype *TP53* gene (TP53^WT^) (*n* = 85); (3) no amplification and gain of *PIK3CA* copy number (PIK3CA^WT^) and TP53^Mutated^ (*n* = 56); and (4) PIK3CA^WT^ and TP53^WT^ (*n* = 54). Only patients with available survival data were included for survival analysis.

The association of mutation grouping with survival was evaluated with Cox regression. The hazard ratios and associated *p*-value are presented with Kaplan Meier curves. Pairwise comparisons utilizing the log-rank test were made between each group. Censoring occurred when a patient was denoted as alive at the end of the observed time period. *p*-values are reported based on a null hypothesis of no effect against a two-sided alternative. Analyses were performed using SAS 9.4 (SAS Institute; Cary, NC, USA).

The downloaded RNA-seq data from HNSCC tumor biopsies were uploaded onto CIBERSORT (https://cibersort.stanford.edu/index.php). CIBERSORT input the RNA-seq data into a matrix of reference gene expression signatures (LM22), which contains 547 genes that distinguish phenotypes of 22 human hematopoietic cell [31,32]. This output was used to estimate the relative proportions of the immune cell type composition of a tumor biopsy. Cell types were summarized by group using the mean, standard deviation, median, and range for continuous variables (Supplemental Table S1). The differences between groups were explored with the omnibus Kruskal−Wallis test due to violations of the normality assumption. Cell types with at least 90% of the subjects in a group having no expression were not tested due to suspect validity of the omnibus test. Pairwise comparisons between groups using Dunn’s Test were performed on cell types that had achieved statistical significance with the omnibus test at the 0.05 level. *p*-values are reported based on a null hypothesis of no effect against a two-sided alternative. Analyses were performed using SAS 9.4 (SAS Institute; Cary, NC, USA).

### 4.2. Mouse Models

Mice were bred to contain the following alleles: a K15 promoter-driven Cre recombinase (K15.CrePR1) [33], a R26Stop^FL^P110* conditional allele that carries a *loxP*-flanked Neo-STOP cassette preceding a constitutively active *PIK3CA* allele (encoding p110α protein) targeted to the *Gt(ROSA)26Sor* locus [34], and a conditional *TP53* gene knockout [35]. Induction of the recombinase activity was achieved by applying 100 μL of RU486 (0.2 μg/μL in 70% ethanol) orally or to the shaved dorsal flank skin of 5 to 12 week-old mice for 5 consecutive days. Afterwards, mice were examined weekly for tumor development (Supplemental Table S2). When tumor size reached 2 cm in any dimension or other humane end points were met, mice were euthanized in accordance with institutional guidelines. Mice were maintained under specific pathogen-free conditions in the vivarium facility of University of Colorado Anschutz Medical Campus (AMC). Animal work was approved by the Institutional Animal Care and Use Committee (IACUC, 00037, 21 Aplril 2020) of University of Colorado AMC (Aurora, CO, USA).

### 4.3. Western Blot and Flow Cytometry

Cells were harvested and lysed using lysis buffer made of 50 mM Tris-base pH 7.5, 150 mM NaCl, 2 mM EDTA, 2 mM Na_3_O_4_V, 4 mM NaF, 1% Triton-X100, 0.1% sodium dodecyl sulfate (SDS), 0.5% sodium deoxycholate, for 30 min on ice. The lysates were centrifuged at 4 °C at 12,000 RPM for 10 min followed by the collection of the supernatant for further analysis. The protein concentrations were calculated using a BCA protein assay kit (Thermo Scientific, Waltham, MA, USA). Each sample (20 μg of protein) was run on SDS-PAGE (Bio-Rad, Hercules, CA, USA) then transferred onto nitrocellulose membrane (Thermo Scientific). Membranes were blocked with 6% milk for 1 h at RT. Then membranes were incubated with specific Abs at various dilutions in 3% milk overnight at 4 °C. The antibodies used were p53 (DO-1) (Santa Cruz, CA, USA) diluted 1:200, PIK3CA (C73F8) (Cell Signaling Technologies, Danvers, MA, USA) diluted 1:1000, and AKT (PAN) (C67E7) (Cell Signaling Technologies) diluted to 1:1000. Membranes were incubated the following day with HRP-conjugated anti-mouse or rabbit Abs diluted at (1:3000) with 3% milk for 2 h at RT. The bands were read using ECL (Thermo Scientific) on a G:Box Chemi-XX6 platform (Syngene, Frederick, MD, USA).

Single cell suspensions were prepared from the spleen of WT B6 mice or tumor-bearing mice and red blood cells (RBC) were lysed with lysing buffer (Sigma Aldrich, St. Louis, MO, USA). Single cell suspensions were prepared from harvested KPPA tumors as follows: finely cutting the tumors with surgical blades into smaller pieces, adding Liberase DL (50 μg/mL) to the diced tumor suspensions, and incubating at 37 °C for 30 min. Then, liberase was neutralized with medium, tumor suspension was filtered through cell strainers (70 µm), and centrifuged at 1500 rpm for 5 min at 4 °C. The single cell suspensions were either stimulated for intracellular cytokine staining (ICS) or analyzed by flow cytometry for the cell surface staining panels. Samples that were to be stimulated were cultured for 4−6 h in the presence of phorbol 12-myristate 13-acetate (PMA) (40 nM) and Ionomycin (650 nM) (LC Laboratories, Woburn, MA, USA), and BD Brefeldin A (BFA) Solution (1×) (BD Biosciences, San Jose, CA, USA, Catalog# 347688) in DMEM medium supplemented with 10% fetal bovine serum, HEPES, antibiotic-antimycotic (100×), and 2 mM glutamine. Cells were stained with 1:1000 LIVE/DEAD Fixable Aqua Dead Cell Stain (Invitrogen, Waltham, MA, USA) in PBS for 20 min in the dark at room temperature (RT). TruStain FcX CD16/32 (BioLegend, San Diego, CA, USA) and Brilliant Stain Buffer Plus (BD Horizon, Franklin Lakes, NJ, USA) were added into each flow panel mixture according to manufacturer’s recommendations. For ICS of IFN-γ and TNF-α, BD Cytofix/CytoPerm buffer kit (BD Biosciences) was used according to the manufacturer’s instructions. For ICS positive control, CD8 T cells were isolated from WT B6 mice and stimulated with anti-CD3/anti-CD28 beads (Dynabeads, Thermo Fisher Scientific) for 3 days. Surface staining and ICS antibodies are listed in Supplemental Table S3. Data were acquired on BD Fortessa and analyzed with FlowJo software V10 (FLOWJO, Ashland, OR, USA).

### 4.4. Histology Analysis

Mouse tumors were fixed in 10% neutral-buffered formalin. Hematoxylin and eosin (H&E) stain and unstained paraffin sections were prepared by the University of Colorado, Denver Research Histology Shared Resource Center. H&E stained paraffin sections were used for histopathological evaluation by a pathologist. For immunofluorescence, 5 μm unstained paraffin sections were deparaffinized by incubating in 61 °C for 10 min. Slides were then immersed in xylene for 5 min, with 2 changes, and hydrated with graded alcohols (100%, 95%, and 70%) for 5 min each. Slides were rinsed in ddH_2_O, immersed in 1× Citrate buffer (pH 6.0) (Invitrogen) and placed in a pressure cooker on high pressure for 5 min. Slides were washed with ddH_2_O, rinsed with 3% H_2_O_2_ for 10 min, and washed with ddH_2_O, and with 0.05% Tris-buffered saline-Tween 20 (TBST). Blocking was performed with 2.5% normal goat serum and 1 h. Afterwards, slides were washed with PBS and then primary antibodies (1:200, chicken-anti-mouse cytokeratin-5 (CK5) (Biolegend, Catalog#905901), 1:100 mouse monoclonal antibody against Vimentin (Vim) (Santa Cruz Biotechnology, Santa Cruz, CA, USA, sc-373717) and 1:10000 4′,6-diamidino-2-phenylindole (DAPI) (Biolegend) were diluted in 1% BSA in PBS, and incubated on the slides overnight at 4 °C. Slides were washed with PBS three times, and 1:400 secondary antibodies, goat-anti-chicken conjugated to Alexa Flour 594 (red) and goat-anti-mouse conjugated to Alexa Flour 488 (green) (ThermoFisher Scientific, Cat# A-11042, Catalog # A-11029, respectively) in 0.05% TBST were added and incubated for 60 min at RT. Finally, slides were observed and evaluated using an Olympus IX83 microscope.

### 4.5. Statistical Analysis of Murine Samples

Statistical analysis was performed using either two-way ANOVA with Tukey’s multiple-comparison test correction or Kruskal−Wallis test with Dunn’s multiple-comparison test correction. GraphPad Prism 8.4.3 software (GraphPad Software, La Jolla, CA, USA) was employed, with significance determined at *p* < 0.05.

## Supplementary Materials

Supplementary materials can be found at https://www.mdpi.com/1422-0067/21/18/6585/s1.
