# Supplementary material for: Deletion of p53 and Hyper-Activation of PIK3CA in Keratin-15+ Stem Cells Lead to the Development of Spontaneous Squamous Cell Carcinoma
Source: Int J Mol Sci. 2020 Sep 9;21(18):6585. doi: 10.3390/ijms21186585 (PMC7554792; doi:10.3390/ijms21186585)
Supplement: Supplementary file 1 [file ijms-21-06585-s001.pdf]

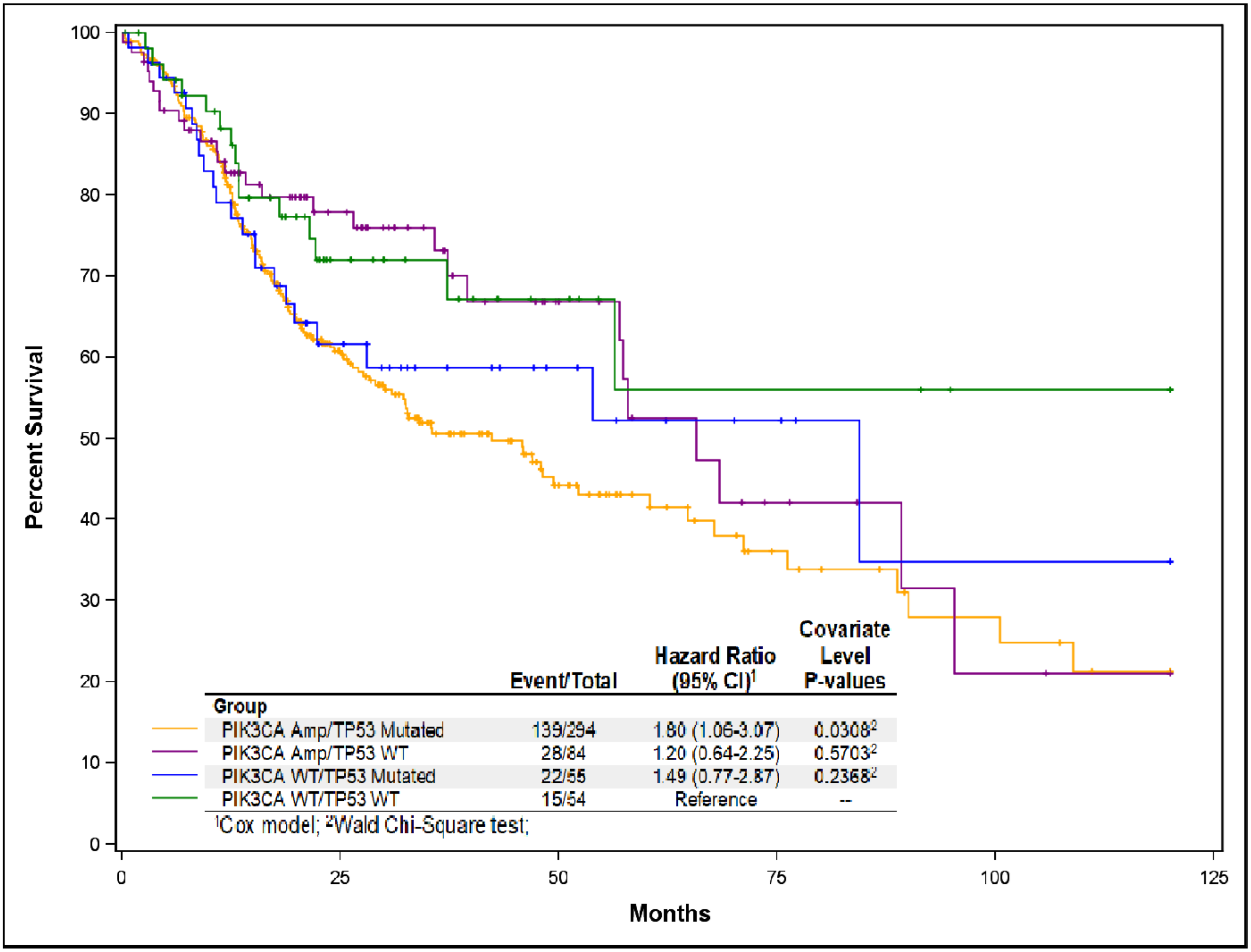

Supplemental Figure 1. Kaplan-Meier overall 10-year survival curves of HNSCC patients in 4 groups ($PIK3CA^{Amp}/TP53^{Mutated}$, $PIK3CA^{Amp}/TP53^{WT}$, $PIK3CA^{WT}/TP53^{Mutated}$, and $PIK3CA^{WT}/TP53^{WT}$). Only patients with available survival data were included for this analysis.

Supplemental Figure 1

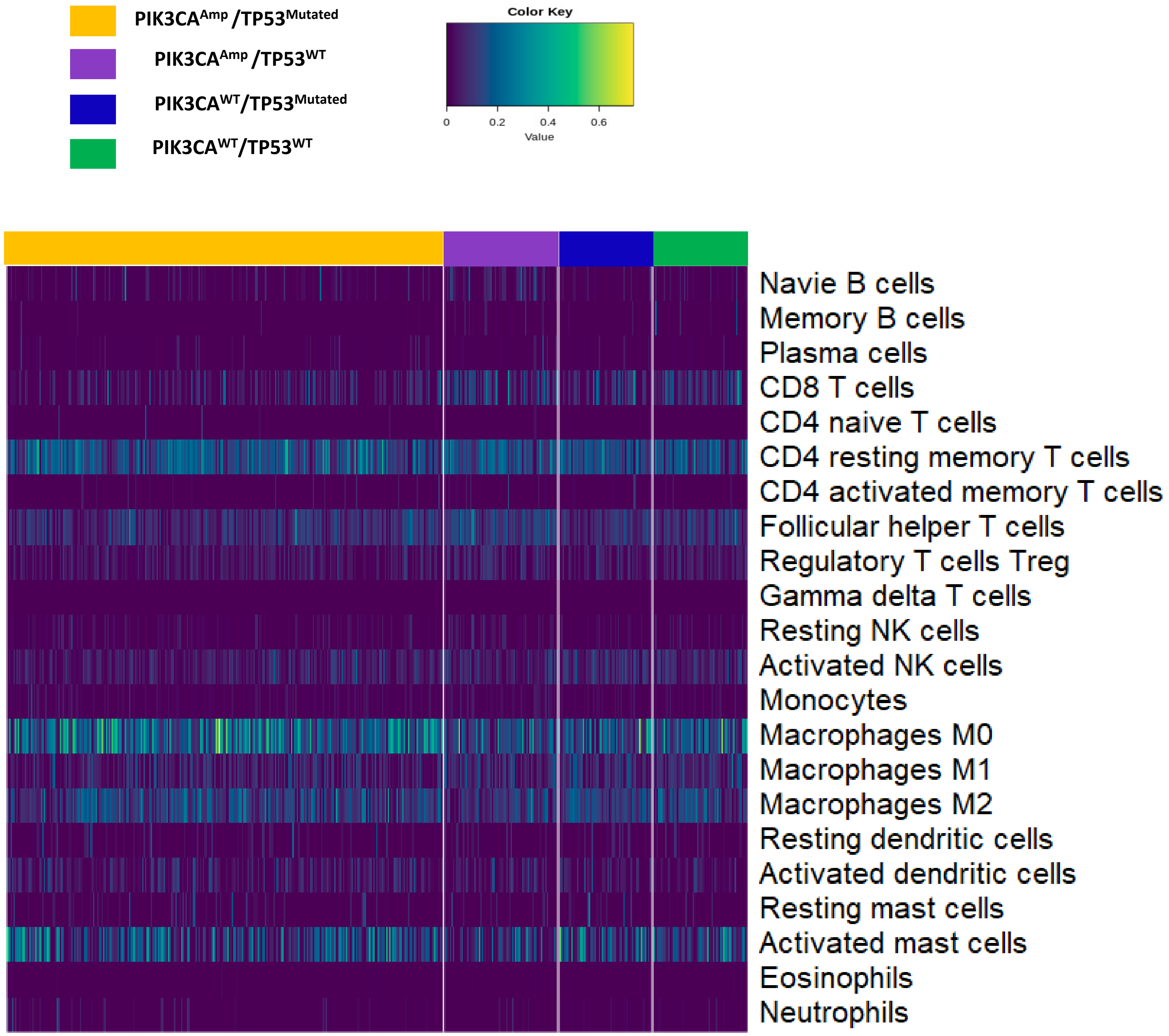

Supplemental Figure 2: Heatmap of composition of 22 immune cell subsets in individual HNSCC patients. Patients were divided into 4 groups (color-coded on top of the graph). Each vertical line represents data from one patient. The results were determined using a custom RNA-Seq leukocyte signature matrix from CIBERSORT (see details in Supplemental Methods). Data were analyzed using log-rank (Mantel-Cox) test.

Supplemental Figure 2

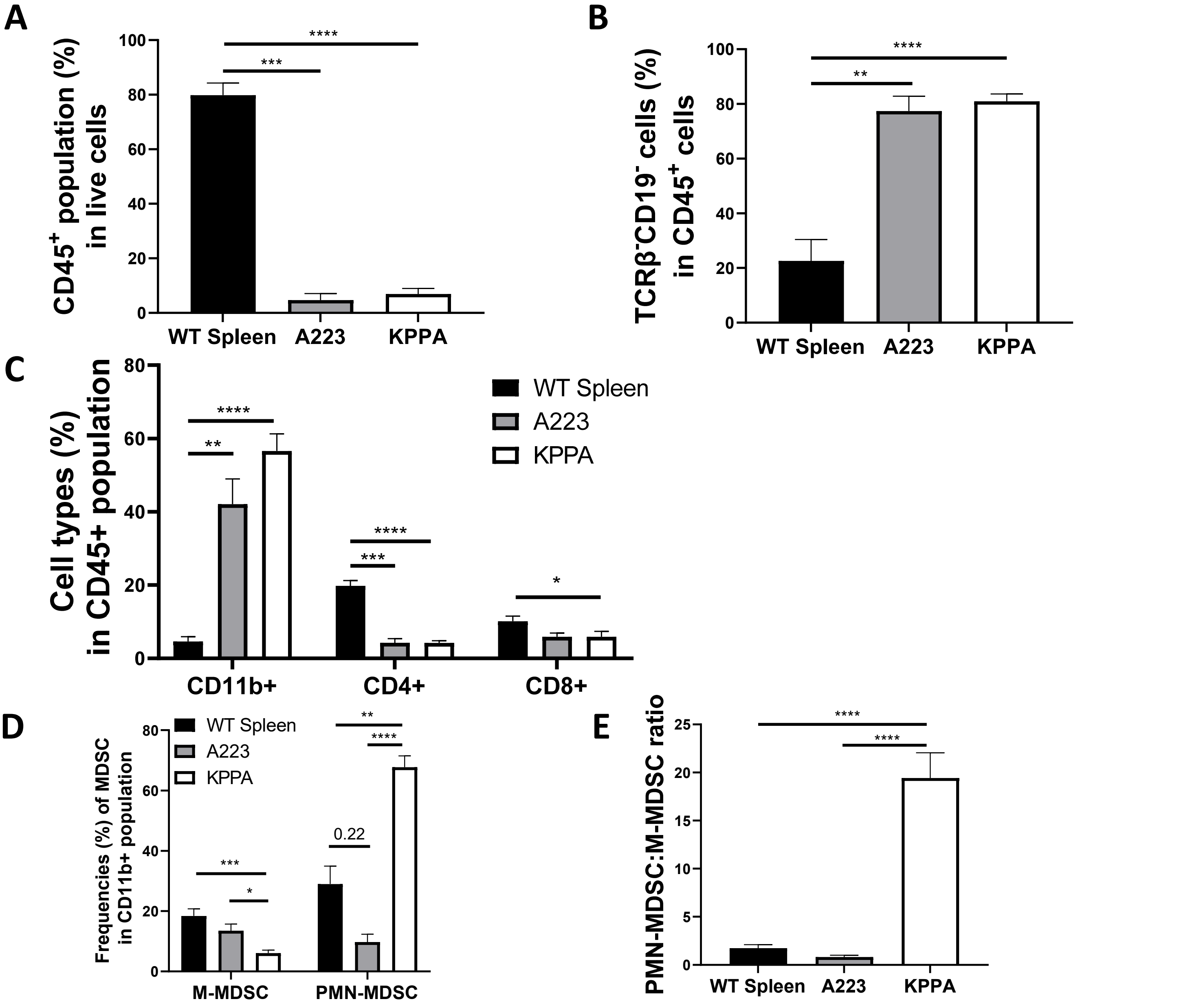

Supplemental Figure 3: Characterization of the immune TME of KPPA tumors. Flow cytometry was performed for WT splenic control (n=13), or the TILs from A223 (n=11) and KPPA (n=25) tumors for all panels shown below. **(A)** Quantification of the percentage of total $CD45^+$ hematopoietic population. The percentage of $CD45^+$ population was significantly greater in WT splenic control (79.82±4.42) compared to A223 (4.63±2.47, p=0.0004) and KPPA (6.93 ±2.05, p≤0.0001) tumors. **(B)** Quantification of the percentage of $CD45^+TCR\beta^-CD19^-$ population (non-T and non-B cells). The percentage of $CD45^+TCR\beta^-CD19^-$ population was significantly lower in WT splenic control (22.61±7.81) compared to A223 (77.44±5.43, p=0.003) and KPPA (80.94±2.69, p≤0.0001) tumors. **(C)** Quantification of the percentage of $CD11b^+$, $CD4^+$, or $CD8^+$ cells in $CD45^+$ population of WT spleen, A223 or KPPA tumors. For $CD11b^+$ cells: WT splenic control (4.62±1.27) was significantly lower than A223 (42.11±6.84, p=0.002) and KPPA (56.57±4.70, p≤0.0001) tumors. For $CD4^+$ cells: A223 (4.26±1.13, p≤0.0001) and KPPA (4.20±0.61, p≤0.0001) tumors were significantly lower than WT splenic control (19.80±1.45). For $CD8^+$ cells: KPPA tumors (5.92±1.44, p=0.018) were significantly lower than WT splenic control (10.14±1.41). **(D)** Quantification of the percentage of M-MDSC vs. PMN-MDSC in indicated groups. There was no difference between WT splenic M-MDSC (18.39±2.35), TB spleen (15.12±1.75), and A223 tumors (13.49±2.22). The percentage of M-MDSC was significantly lower in KPPA tumors (6.07±1.02) than WT spleen (p≤0.0001) or A223 tumors (p=0.018). The percentage of PMN-MDSC was significantly higher in KPPA tumors (67.74±3.72) than WT spleen (29.00±5.95, p=0.002) or A223 tumors (9.78±2.53, p≤0.0001). **(E)** The ratio of PMN-MDSCs vs. M-MDSCs in indicated groups. PMN-MDSC vs. M-MDSC ratio in KPPA tumors (19.42±1.64) was significantly higher than WT splenic control (1.74±0.37, p≤0.0001) and A223 (0.81±0.18, p≤0.0001). Data were analyzed using Kruskal-Wallis test *p<0.05, **p<0.01, ***p<0.001, ****p<0.0001, with Dunn's multiple-comparison test correction.

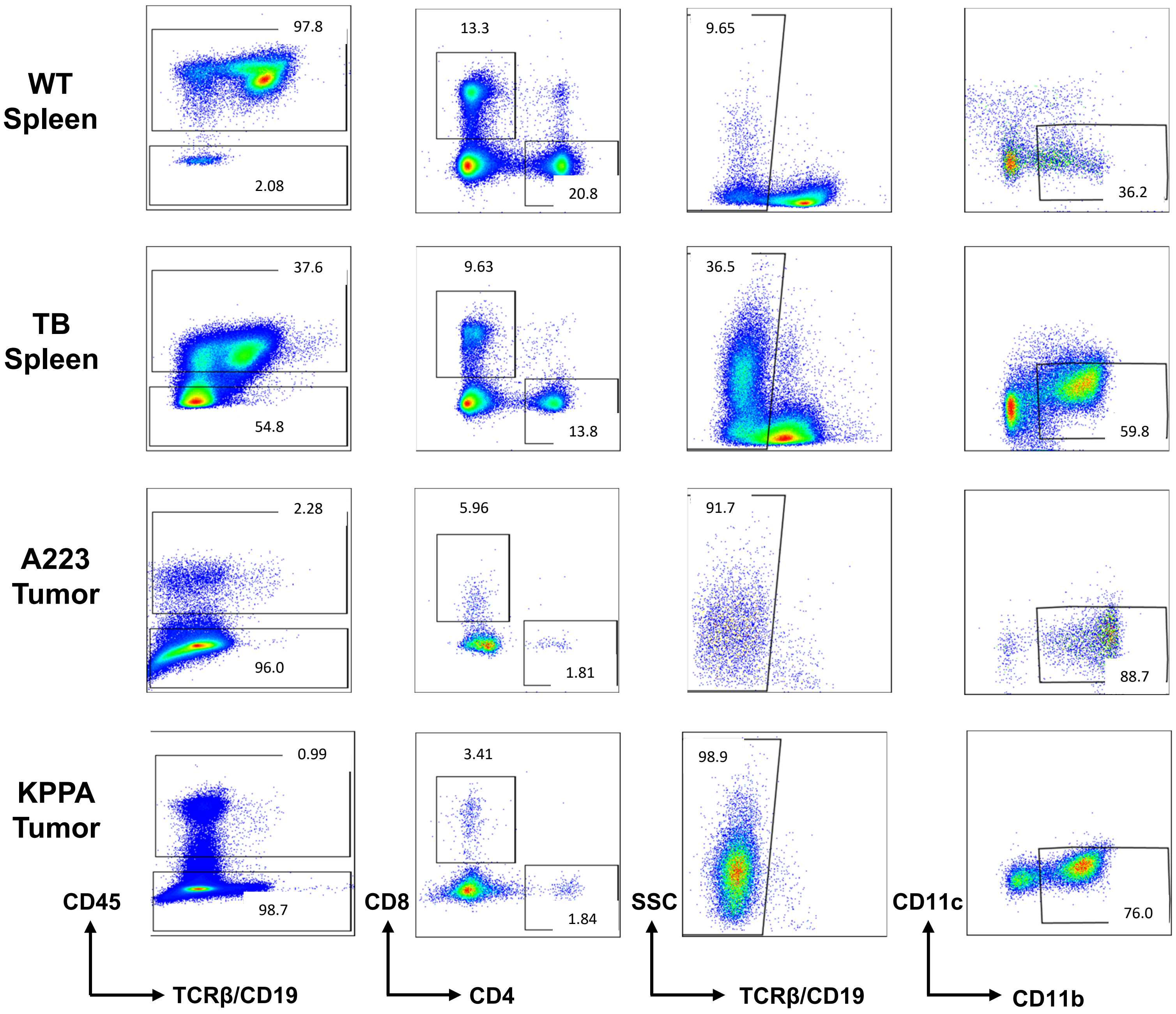

Supplemental Figure 4: Representative flow plots of different cell types analyzed. Flow plots of $CD45^+$ cells, $CD8^+$ T cells, $CD4^+$ T cells, non-T/non-B cells, and $CD11b^+$ cell populations in WT spleen, TB spleen, A223 tumors and KPPA tumors.

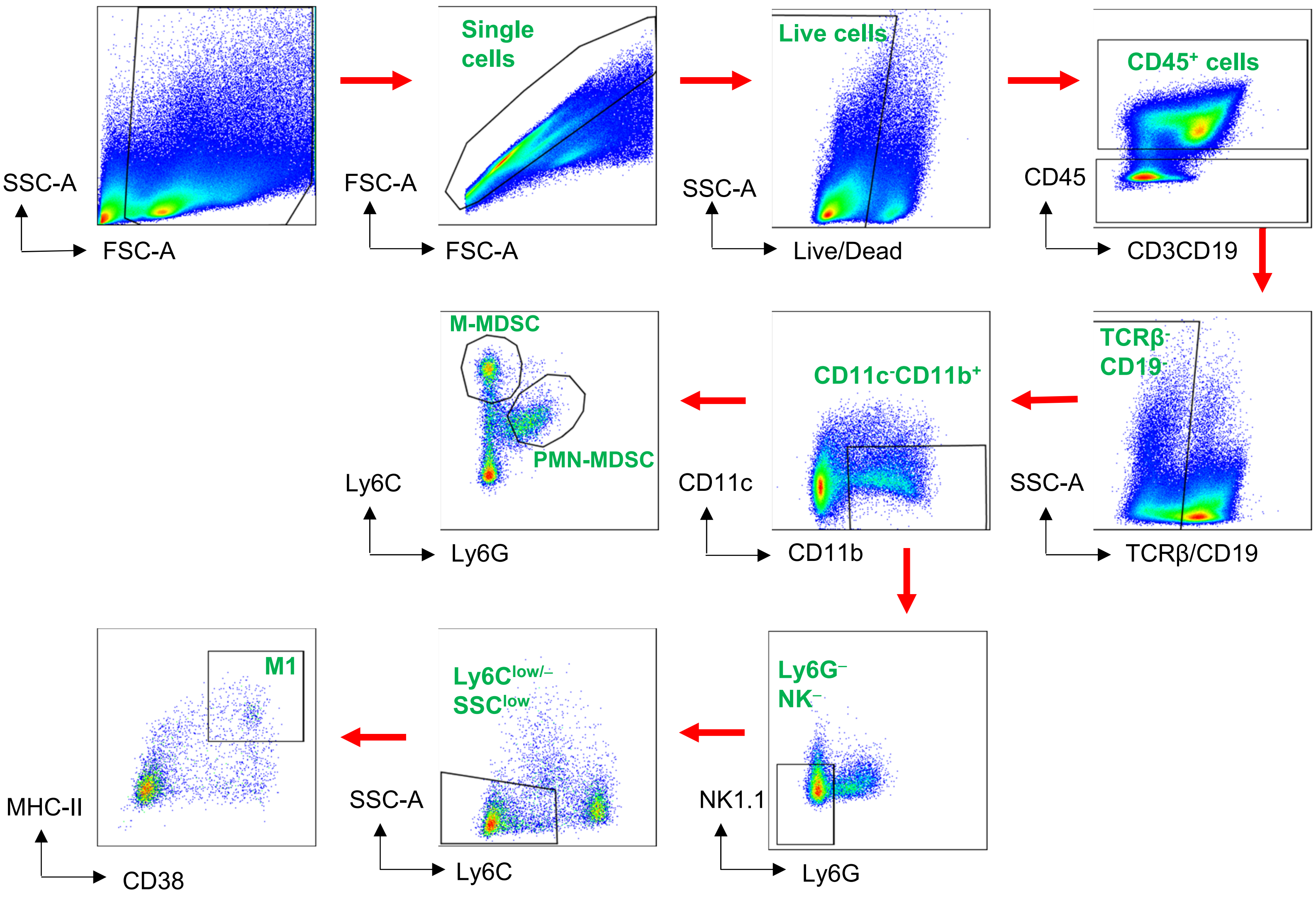

Supplemental Figure 5: Monocyte/Macrophage gating strategy. M-MDSC and PMN-MDSC were gated following previously published work (PMID: 27381735 and PMID: 25504825). Classically activated M1 population was gated following previously published work (PMID: 22213571, PMID: 26699615, PMID: 22822406, PMID: 21813021).

Supplemental Table 1: Composition of 22 immune cell types in 4 different groups of HNSCC patients

	Mutation Category					
**Variables**	**PIK3CA WT<br>TP53 WT<br>(N=54)**	**PIK3CA WT<br>TP53 Mutated<br>(N=56)**	**PIK3CA Amp<br>TP53 WT<br>(N=85)**	**PIK3CA Amp<br>TP53 Mutated<br>(N=294)**	**P-value**	**Overall**
**Cell Composition**						
Naïve B cells[5,7]	1.59 (2.31)<br>0.71 (0, 1.72)	1.02 (1.66)<br>0.28 (0, 1.4)	3.19 (4.39)<br>1.26 (0.33, 4.42)	1.4 (3.04)<br>0.4 (0, 1.33)	<.0001[1]	1.69 (3.2)<br>0.54 (0, 1.78)
Memory B cells	1.29 (4.42)<br>0 (0, 0)	0.17 (0.76)<br>0 (0, 0)	0.6 (1.59)<br>0 (0, 0)	0.1 (0.64)<br>0 (0, 0)	--	0.33 (1.74)<br>0 (0, 0)
Plasma cells	0.43 (0.85)<br>0.04 (0, 0.62)	0.53 (1.25)<br>0 (0, 0.46)	0.74 (1.73)<br>0.25 (0, 0.78)	0.71 (1.39)<br>0.24 (0, 0.84)	0.0765[1]	0.66 (1.39)<br>0.17 (0, 0.75)
CD8 T cells[4,7]	8.97 (7.86)<br>7.21 (3.24, 12.79)	6.23 (7.63)<br>3.4 (0, 9.83)	8.22 (8.12)<br>7.03 (0, 12.62)	3.77 (5.32)<br>1.29 (0, 6.08)	<.0001[1]	5.4 (6.79)<br>3.19 (0, 8.5)
Naïve CD4 T cells	0 (0)<br>0 (0, 0)	0 (0.01)<br>0 (0, 0)	0.04 (0.38)<br>0 (0, 0)	0.18 (1.48)<br>0 (0, 0)	--	0.12 (1.16)<br>0 (0, 0)
Resting memory CD4 T cells	14.26 (8.31)<br>15.01 (7.45, 20.73)	14.58 (6.49)<br>15.15 (10.21, 17.49)	15.01 (7.16)<br>14.65 (9.45, 20.36)	16.01 (8.63)<br>15.55 (9.94, 21.49)	0.6161[1]	15.48 (8.14)<br>15.28 (9.73, 20.82)
Activated memory CD4 T cells	0.44 (1.06)<br>0 (0, 0)	0.43 (1.85)<br>0 (0, 0)	0.6 (1.78)<br>0 (0, 0.2)	0.3 (1.16)<br>0 (0, 0)	--	0.38 (1.37)<br>0 (0, 0)
Follicular helper T cells[5,7]	9 (4.9)<br>7.68 (6.3, 11.15)	7.98 (4.58)<br>7.6 (4.21, 10.23)	11.2 (5.29)<br>10.27 (7.85, 14.15)	7.9 (5.16)<br>7.44 (3.85, 11.04)	<.0001[1]	8.61 (5.22)<br>7.97 (4.84, 11.77)
Regulatory T cells	4.57 (3.01)<br>4.62 (2.01, 6.49)	3.7 (3.42)<br>2.82 (0.75, 6.11)	5.13 (4.02)<br>4.43 (1.48, 8.18)	3.59 (3.12)<br>3.25 (1.09, 5.31)	0.0033[1]	3.98 (3.36)<br>3.49 (1.23, 5.96)
Gamma delta T cells	0 (0.01)<br>0 (0, 0)	0 (0)<br>0 (0, 0)	0 (0)<br>0 (0, 0)	0 (0.06)<br>0 (0, 0)	--	0 (0.04)<br>0 (0, 0)
Resting NK cells	1.25 (1.93)<br>0 (0, 1.97)	0.88 (1.47)<br>0 (0, 1.3)	1.8 (2.46)<br>0.46 (0, 3.09)	1.21 (1.8)<br>0.29 (0, 2.04)	0.0961[1]	1.28 (1.93)<br>0.15 (0, 2.16)
Activated NK cells[4,6]	6.9 (4.91)<br>6.58 (3.31, 8.92)	6.91 (4.71)<br>6.31 (3.04, 10.58)	5.5 (4.03)<br>4.43 (2.91, 7.25)	4.92 (3.89)<br>4.27 (2.12, 7.11)	0.0013[1]	5.47 (4.2)<br>4.65 (2.48, 7.84)
Monocytes[7]	1.31 (1.38)<br>0.84 (0, 2.24)	1.11 (1.27)<br>0.72 (0, 1.68)	1.76 (1.66)<br>1.6 (0.22, 2.54)	1.2 (1.63)<br>0.67 (0, 1.98)	0.0081[1]	1.3 (1.58)<br>0.9 (0, 2.05)
Macrophages M0[4,7]	15.25 (12.32)<br>11.93 (5.71, 23.14)	16.43 (13.39)<br>12.6 (7.4, 23.71)	13.93 (11.23)<br>11.47 (7.18, 17.97)	21.07 (14.08)<br>19.02 (9.31, 30.04)	<.0001[1]	18.66 (13.66)<br>15.65 (8.18, 26.49)
Macrophages M1[4,7]	8.17 (5.62)<br>7.83 (4.12, 11.49)	6.71 (4.97)<br>6.37 (2.63, 10.24)	6.92 (5.17)<br>6.48 (3.49, 9.14)	5.35 (5.05)<br>4.21 (0.75, 8.81)	0.0002[1]	6.09 (5.21)<br>5.2 (1.58, 9.41)
Macrophages M2	10.27 (7.09)<br>9.49 (5.17, 15.44)	12.66 (5.35)<br>12.94 (8.04, 15.77)	10.23 (6.74)<br>9.63 (5.04, 13.83)	11.78 (7.73)<br>10.46 (5.76, 17.34)	0.0655[1]	11.44 (7.29)<br>10.51 (5.87, 15.78)
Resting DCs	1.14 (2.38)<br>0 (0, 0.56)	1.17 (2.26)<br>0 (0, 1.34)	1.22 (2.31)<br>0 (0, 1.51)	1.36 (3.38)<br>0 (0, 0.9)	0.7573[1]	1.29 (3)<br>0 (0, 1.01)
Activated DCs[4]	2.52 (3.26)<br>1.53 (0, 3.47)	3.87 (3.93)<br>2.52 (0.78, 6.3)	3.77 (4.26)<br>2.67 (0.07, 5.59)	4.33 (4.42)<br>3.16 (0.49, 6.51)	0.0364[1]	3.98 (4.25)<br>2.72 (0.33, 6.08)
Resting Mast cells	0.4 (1.47)<br>0 (0, 0)	1.62 (4.89)<br>0 (0, 0.1)	1.01 (2.43)<br>0 (0, 0)	1.09 (2.93)<br>0 (0, 0)	--	1.06 (3.03)<br>0 (0, 0)
Activated mast cells	11.79 (10.9)<br>10.61 (2.84, 14.5)	13.4 (12.89)<br>12.51 (2.87, 19.59)	8.63 (9.47)<br>4.85 (0.59, 15.14)	12.96 (11.95)<br>10.66 (2.04, 20.51)	0.0302[1]	12.13 (11.64)<br>9.37 (2.29, 18.91)
Eosinophils	0.06 (0.27)<br>0 (0, 0)	0.01 (0.08)<br>0 (0, 0)	0.06 (0.24)<br>0 (0, 0)	0.1 (0.34)<br>0 (0, 0)	--	0.08 (0.3)<br>0 (0, 0)
Neutrophils	0.39 (0.83)<br>0 (0, 0.32)	0.56 (0.82)<br>0.01 (0, 0.87)	0.46 (1.27)<br>0 (0, 0.09)	0.68 (1.83)<br>0 (0, 0.41)	0.0874[1]	0.6 (1.57)<br>0 (0, 0.49)

Supplemental Table 1. Results are presented in the following format: mean (standard deviation) median (Q1, Q3). The p-values in this table are omnibus tests of the null hypothesis of no difference between the mutation groups.

1: Kruskal-Wallis Test: Pairwise Comparisons using Dunn's Test performed for Naïve B cells, Plasma Cells, CD8 T Cells, Follicular helper T cells, Regulatory T cells, Resting NK cells, Activated NK cells, Monocytes, Macrophages M0, Macrophages M1, Macrophages M2, Activated DCs, Activated mast cells, Neutrophils.

Cell types with the following annotations indicate a rejection at the 0.05 level (*p<0.05), groups that are statistically significant are labeled in red fonts:

2: $PIK3CA^{WT}/TP53^{WT}$ vs. $PIK3CA^{WT}/TP53^{Mutated}$
3: $PIK3CA\ ^{WT}/TP53^{WT}$ vs. $PIK3CA^{Amp}/P53^{WT}$
4: $PIK3CA^{WT}/TP53^{WT}$ vs. $PIK3CA^{Amp}/P53^{Mutated}$
5: $PIK3CA^{WT}/TP53^{Mutated}$ vs. $PIK3CA^{Amp}/TP53^{WT}$
6: $PIK3CA^{WT}/TP53^{Mutated}$ vs. $PIK3CA^{Amp}/TP53^{Mutated}$
7: $PIK3CA^{Amp}/TP53^{WT}$ vs. $PIK3CA^{Amp}/TP53^{Mutated}$

Supplemental Table 2: Kinetics of tumor development in K15Cre(+)PIK3CA$^{c/c}$TP53$^{f/f}$ mice

Mouse ID	Sex	DOB	Genotype	Tumor development Months after RU486	Tumor Histology
76P	F	3/21/2109	Cre^{PR1}PIK3CA$^{C/C}$TP53$^{f/f}$	3.06	poly-differentiated SCC
78P	M	3/21/2109	Cre^{PR1}PIK3CA$^{C/C}$TP53$^{f/f}$	3.53	angioma with thrombus formation
89P	M	2/13/2019	Cre^{PR1}PIK3CA$^{C/C}$TP53$^{f/f}$	2.2	SCC
121P	F	5/24/2019	Cre^{PR1}PIK3CA$^{C/C}$TP53$^{f/f}$	3.76	adenosarcoma
126P	F	5/24/2019	Cre^{PR1}PIK3CA$^{C/C}$TP53$^{f/f}$	3.26	angioma with thrombus formation
127P	F	5/24/2019	Cre^{PR1}PIK3CA$^{C/C}$TP53$^{f/f}$	3.26	SCC
128P	F	5/24/2019	Cre^{PR1}PIK3CA$^{C/C}$TP53$^{f/f}$	3.26	NA
148P	M	7/8/2019	Cre^{PR1}PIK3CA$^{C/C}$TP53$^{f/f}$	3.86	NA
150P	M	7/8/2019	Cre^{PR1}PIK3CA$^{C/C}$TP53$^{f/f}$	1.16	angioma with thrombus formation
156P	F	7/14/2019	Cre^{PR1}PIK3CA$^{C/C}$TP53$^{f/f}$	0.5	angioma with thrombus formation
158P	F	7/14/2019	Cre^{PR1}PIK3CA$^{C/C}$TP53$^{f/f}$	3.76	NA
164P	M	7/17/2019	Cre^{PR1}PIK3CA$^{C/C}$TP53$^{f/f}$	2.8	NA
168P	M	7/17/2019	Cre^{PR1}PIK3CA$^{C/C}$TP53$^{f/f}$	2.8	NA
170P	M	8/4/2019	Cre^{PR1}PIK3CA$^{C/C}$TP53$^{f/f}$	5	NA

Supplementary Table 3: Antibodies used in this study

Antibody	Fluorophore	Company	Catalog	Clone	Concentration
CD11c	PerCP/Cy5.5	BioLegend	117327	N418	1μg/mL
PD-L1	BV786	BD Bioscience	741014	MIH5	1μg/mL
MHCII	BV711	BioLegend	107643	M5/114/15/2	0.25μg/mL
CD19	Brilliant Violet 605	BioLegend	115539	6D5	1μg/mL
Ly6C	BV421	BioLegend	128031	HK1.4	1μg/mL
Ly-6G	APC/Cy7	BioLegend	127623	1A8	1μg/mL
CD11b	Alexa Fluor 700	BioLegend	101222	M1/70	1μg/mL
CD223 (LAG-3)	BV785	BioLegend	125219	C9B7W	1μg/mL
CD279 (PD-1)	BV711	BioLegend	135231	29F.1A12	1μg/mL
CD366 (Tim-3)	APC	BioLegend	134007	B8.2C12	1μg/mL
TCR beta	BV605	BioLegend	109241	H57-597	1μg/mL
CD4	BV421	BioLegend	100563	RM4-5	1μg/mL
CD8a	Alexa Fluor 700	BioLegend	100729	53-6.7	1μg/mL
CD45	BUV395	BD Bioscience	564279	30-F11	1μg/mL
TNFalpha	BV650	BD Bioscience	563943	mp6-xt22	1μg/mL
IFN gamma	PE	eBioscience	12-7311-41	XMG1.2	1μg/mL
